# Insular atrophy at the prodromal stage of dementia with Lewy bodies: a VBM DARTEL study

**DOI:** 10.1038/s41598-017-08667-7

**Published:** 2017-08-25

**Authors:** Daniel Roquet, Vincent Noblet, Pierre Anthony, Nathalie Philippi, Catherine Demuynck, Benjamin Cretin, Catherine Martin-Hunyadi, Paulo Loureiro de Sousa, Frédéric Blanc

**Affiliations:** 1 ICube, UMR 7357, CNRS, Université de Strasbourg, Fédération de Médecine Translationnelle de Strasbourg, Strasbourg, France; 2 CMRR (Centre Mémoire de Ressources et de Recherche), University Hospital of Strasbourg, Strasbourg, France

## Abstract

Diffuse atrophy including the insula was previously demonstrated in dementia with Lewy bodies (DLB) patients but little is known about the prodromal stage of DLB (pro-DLB). In this prospective study, we used SPM8-DARTEL to measure gray matter (GM) and white matter (WM) atrophy in pro-DLB patients (n = 54), prodromal Alzheimer’s disease (pro-AD) patients (n = 16), DLB patients at the stage of dementia (mild-DLB) (n = 15), and Alzheimer’s disease patients at the stage of dementia (mild-AD) (n = 28), and compared them with healthy elderly controls (HC, n = 22). Diminished GM volumes were found in bilateral insula in pro-DLB patients, a trend to significance in right hippocampus and parahippocampal gyrus in pro-AD patients, in left insula in mild-DLB patients, and in medial temporal lobes and insula in mild-AD patients. The comparison between prodromal groups did not showed any differences. The comparison between groups with dementia revealed atrophy around the left middle temporal gyrus in mild-AD patients. Reduced WM volume was observed in mild-DLB in the pons. The insula seems to be a key region in DLB as early as the prodromal stage. MRI studies looking at perfusion, and functional and anatomical connectivity are now needed to better understand the role of this region in DLB.

## Introduction

Dementia with Lewy bodies (DLB) is one of the main etiologies of neurodegenerative dementia after Alzheimer’s disease (AD). DLB accounts for 15% to 20% of cases according to neuropathologically defined cases^1^. The diagnostic classification of DLB is based on revised consensus criteria, the core diagnostic features of DLB being (1) recurrent visual hallucinations, (2) fluctuations in cognition or alertness, and (3) spontaneous motor features of parkinsonism^1^. The presence of 2 or 3 of these core signs is sufficient for a diagnosis of probable DLB^1^. The same definition of DLB has been proposed by DSM V at the stage of dementia (also named major neurocognitive disorder) but also at the stage of mild cognitive impairment (MCI) (also named mild neurocognitive disorder or prodromal stage)^2^. Distinguishing DLB from AD is difficult because of overlapping clinical and neuropathological features between the two conditions, but also because specific symptoms of DLB such as hallucinations or cognitive fluctuations are not spontaneously described by the patient and the caregiver. The accurate differentiation of DLB and AD, however, is particularly important as: (1) the neuropathological lesions are different even if they can be associated^3^; (2) DLB patients exhibit sensitivity to neuroleptics with worsening of clinical status; and (3) DLB patients have a differing prognosis compared to AD patients^4^, but have a better response to cholinesterase inhibitors^5^.

The diagnostic challenge becomes particularly salient in the early stages^6^. In contrast to AD, where there are significant advances in the classification and definition of prodromal AD (pro-AD)^7^, the diagnostic classification of pro-DLB remains in its infancy although a prodromal phase of DLB has now been demarcated in DSM-V as mild neurocognitive disorder of Lewy body disease^2^ and preliminary descriptions of pro-DLB criteria have recently been published^8^. Broadly, pro-DLB patients can be defined as those who meet the revised diagnostic criteria for DLB but fit the criteria for MCI^6^ instead of dementia^1^.

Pro-DLB has been described with a different cognitive pattern from pro-AD^9, 10^: at the early stage of the disease, DLB patients have more visuospatial and less fluency deficits than AD, and AD patients have more memory storage impairment than DLB^10, 11^. These findings are in keeping with the cognitive profiles of AD and DLB patients when the dementia becomes manifest^12^, although the neuropsychological pattern in prodromal DLB (pro-DLB) has been reported as more heterogeneous than in pro-AD^11^.

Nevertheless, early identification of DLB, particularly in the prodromal phase (i.e., pro-DLB), will be highly relevant to the development and testing of future disease modifying treatments and thus there is an urgent need to develop viable and sensitive biomarkers that can detect DLB in its early stages. Furthermore, determination of early biomarkers in DLB is necessary to guide the operationalization of future consensus criteria for pro-DLB^8^.

Structural neuroimaging represents one potential biomarker area: in particular voxel-based morphometry (VBM) is a classical approach to detect brain atrophy. In AD compared to healthy elderly controls, VBM studies described atrophy of the entorhinal cortex and hippocampus as early as the prodromal stage of AD^13–17^, which was also shown to be a good predictor of cognitive decline and conversion to dementia^15, 18–21^. In AD at the stage of dementia, in addition to the medial temporal lobe, other atrophies were reported in temporal, parietal, posterior cingulate and frontal cortices as well as insula and rolandic operculum^14, 22–24^. Previous VBM studies in DLB at the stage of dementia (mild-DLB) have demonstrated heterogeneous results. The Newcastle team showed a diminished gray matter (GM) volume in the lateral temporal lobes, frontal lobe, insulae, and precuneus^25^. On the other hand, the Mayo Clinic team showed atrophy in midbrain, substantia innominata, hypothalamus and right insula^23^. A voxel-wise meta-analysis on cortical atrophy in mild-DLB patients found bilateral insula and basal ganglia atrophy^26^. We recently demonstrated in a multicentre study that the cortical thickness of pro-DLB patients is diminished in the right anterior part of the insula^22^. This first result is of high interest since patients at the early stage of the disease frequently present neurovegetative disorders such as constipation, orthostatic dizziness, or increased saliva^27^, which could be linked to insula dysfunction^28^. Pro-DLB patients also frequently have fluctuations and hallucinations that could also be linked to the insula^29, 30^. In contrast, parkinsonism is rarely obvious at the beginning of DLB, and such symptoms are known to be linked to basal ganglia and particularly the substantia nigra. While cortical thickness and VBM are both related to gray matter, they are not fully consistent^31^. Moreover, we aimed to compare patients that have been screened in a homogeneous way.

When assessing white matter (WM) integrity, a meta-analysis^32^ showed that AD was mainly characterized by WM medial temporal lobe atrophy, although monocentre studies describe reduced WM in other parts of the brain such as the parietal^33, 34^ and frontal^35^ lobes, together with the inferior longitudinal fasciculus^33^ and corpus callosum^32, 33^. To our knowledge, compared to GM atrophy, VBM-based WM atrophy has been less investigated in DLB. Lee *et al*.^36^ reported a loss of WM at the stage of dementia in mainly the posterior areas, but at an uncorrected statistical threshold. Only two studies have compared WM between DLB and AD: although the first study^34^ did not showed any reduction of WM volume in DLB, the second study^37^ showed an atrophy in DLB in the dorsal midbrain, pons and the cerebellum. No investigations have been performed yet at the prodromal stage.

Therefore, the aim of this study was to investigate, in a monocentre study, GM and WM atrophy patterns in pro-DLB and in mild-DLB patients. To this end, GM and WM volumes were compared to those of healthy elderly controls (HC) and those of AD patients at either the prodromal stage (pro-AD) or the mild dementia stage (mild-AD). According to patterns of atrophy in both the prodromal and dementia stages in AD and DLB, we made the following assumptions. First, we hypothesized that, compared to HC, pro-DLB patients would have GM atrophy predominantly in the anterior insula, but pro-AD patients would have GM atrophy in the entorhinal cortex and the hippocampus. As a consequence, we expected that the comparisons of the two prodromal groups of patients would provide a similar pattern of atrophy. At the stage of dementia, we hypothesized that the GM atrophy of insula in DLB patients would enlarged to the entire insula, and expand to basal ganglia and frontal cortex, whereas the medial temporal lobe atrophy in AD patients would spread to insula, temporal, posterior cingulate and frontal cortices. Accordingly, we expected that the comparisons of DLB and AD patients at the stage of dementia would not lead to statistical differences in the insula. If present, we also expected slight WM atrophy in the brainstem for prodromal DLB patients and in the medial temporal lobe for prodromal AD patients, which would enlarge at the stage of dementia.

## Material and Methods

### Participants

Information about the goals of the prospective longitudinal AlphaLewyMA study is freely available on the Clinical Trials web site (http://clinicaltrials.gov/ct2/show/NCT01876459). Briefly, the primary end point was to assess synuclein in the cerebrospinal fluid, and the secondary end point, which concerned the present study, was to create biomarker for DLB and AD using MRI. One hundred forty-four patients with either DLB or AD were consecutively recruited from the Memory Resources and Research Center of the University Hospital of Strasbourg, France, together with 22 elderly healthy controls. Nine participants were excluded due to motion during the MRI acquisition (n = 9). The remaining 135 patients were classified according to their diagnosis and the level of their cognitive deficit. Consequently, the study comprised 54 patients with pro-DLB (pro-DLB group), 15 patients with DLB at the dementia stage (mild-DLB group), 16 patients with pro-AD (pro-AD group), 28 patients with AD at the dementia stage (mild-AD group), and 22 healthy elderly controls (HC group). Demographic data are presented in Table 1. Patients presenting both DLB and AD clinical features were excluded from the analysis.

**Table 1:** Characteristics of patients and healthy subjects.

Characteristic	pro-DLB	mild-DLB	pro-AD	mild-AD	HC
Participants	54	15	16	28	22
Female	31	7	5	19	12
Age, years (SD)	69.3 (9.0)	74.3 (10.4)^*^	75.3 (9.2)^**^	74.1 (8.8)^**^	65.6 (9.2)
MMSE score (SD) (max 30)	27.6 (1.4)	20.7 (3.4)	27.1 (1.4)	19.3 (3.3)	29.0 (1.0)
Participants with visual hallucinations	23	9	0	2	0
Participants with fluctuations	32	8	0	4	0
Participants with parkinsonism	40	10	3	7	0

pro-DLB, dementia with Lewy bodies at the prodromal stage; mild-DLB, dementia with Lewy bodies at the stage of dementia; pro-AD, Alzheimer’s disease at the prodromal stage; mild-AD, Alzheimer’s disease at the stage of dementia; HC, healthy controls. MMSE: mini mental state examination. SD: standard deviation.

*p < 0.05; **p < 0.01 com*p*ared to HC resulting from ANOVA and post hoc analysis.

The five groups were examined by clinicians with expertise (tertiary center) in dementia to perform a complete anamnesis and medical examination. Using the Unified Parkinson’s Disease Rating Scale (UPDRS) III score^38^, akinesia, rigidity, and tremor at rest were rated from 0 to 4 (0: no symptoms to 4: serious impairment). Fluctuations were assessed with the Mayo Clinic Fluctuations scale (patients with a score higher than or equal to two were considered as having fluctuations) and the Newcastle-upon-Tyne Clinician Assessment of Fluctuation scale. Cognitive functions were evaluated, and the cognitive profile of the pro-DLB patients of the AlphaLewyMA group has been reported by Kemp *et al*.^39^.

An etiologic diagnosis of the neurocognitive disorder for each patient was made using Dubois’ criteria for pro-AD (i.e., MCI AD) and mild-AD^7^, and McKeith’s criteria (probable DLB, i.e. at least two core symptoms) for mild-DLB^1^. Pro-DLB patients were defined as patients with MCI (according to Petersen criteria^40^), preservation of independence (assessed by the Instrumental Activities of Daily Living [IADL]) and by McKeith’s criteria (meeting probable DLB criteria except presence of dementia)^1^. All patients had formal assessment of their diagnosis by three independent expert clinicians. Patients with concomitant AD and DLB, i.e., meeting both McKeith’s (for probable DLB) and Dubois’ criteria, were also excluded.

The control group consisted in elderly healthy and cognitively intact (no MCI) subjects who were recruited via advertisements in local community newsletters in Strasbourg, and via the listing of controls of the local clinical investigation center (“*Centre d’Investigation Clinique*”) in charge of any type of medical research of the University Hospital of Strasbourg. In the Memory Resources and Research Center, controls underwent similar clinical and cognitive assessments than patients to exclude any who may have had occult MCI or dementia.

Exclusion criteria for participation in the study included contraindications for MRI, history of alcohol/substance misuse, evidence suggesting alternative neurological or psychiatric explanations for their symptoms/cognitive impairment, focal brain lesions on brain imaging, and the presence of other severe or unstable medical illness.

The study was approved by the local Ethics Committee (*Comité de Protections des Personnes*, Est IV, Strasbourg, France), and all experiments were performed according to the guidelines and regulations provided by this committee. Controls and patients gave written informed consent.

### Data acquisition

A 3D MPRAGE T1-weighted image was acquired on a Siemens Verio 3 T scanner equipped with a 32-channel head coil (Siemens, Erlangen Germany). Parameters were: sagittal orientation; repetition time = 1900 ms; echo time = 2.53 ms; inversion time = 900 ms; flip angle = 9°; imaging matrix 192 × 192 × 176; 1 mm^3^ isotropic voxel; acquisition time = 7 min 38 s. The image was acquired such that the axial slice orientation was aligned with the AC-PC line.

### Data processing

Images were processed using SPM8 (Welcome Department of Cognitive Neurology, London, UK). According to the algorithm of the *New Segment* toolbox of SPM8, images were first segmented into five tissue probability maps in the native space. Two GM DARTEL templates were then computed from GM and WM probability maps of either a subset that included the pro-DLB, pro-AD and HC groups, or another subset that included the mild-DLB, mild-AD and HC groups. The GM and WM probability maps of each participant were spatially normalized to the Montreal National Institute (MNI) space according to the transformation parameters from the corresponding DARTEL template (since healthy participants were including in the two subsets, the normalization was processed twice according to each DARTEL template). During this procedure, images were modulated, then smoothed with a Gaussian filter of full width at half maximum of 8 mm.

### Statistical analysis

Patients with either MCI or dementia were analyzed separately, i.e. a first set of analyses compared the pro-DLB, pro-AD and HC groups, whereas another compared the mild-DLB, mild-AD and HC groups.

Analyses were performed on Matlab (R2014b, Mathworks, Natick, MA). The distribution of gender and age values between groups was assessed by chi-squared tests and a one-way ANOVA, respectively. Prior to ANOVA, homoscedasticity and normality of the distribution of age values were verified by Levene’s test and Lilliefors tests to ensure that our conditions really met the assumptions of ANOVA as a parametric test. Post-hoc analyses were performed using Tukey–Kramer multi-comparison test.

Using the *randomize* function of FSL 5.0 (FMRIB Analysis Group, Oxford, UK), non-parametric permutation tests with threshold-free cluster enhancement were performed to compare GM and WM volumes between groups, by running 5,000 permutations with variance smoothing of 8 mm. Age, gender and total intracranial volume were regarded as regressors of non-interest. The total intracranial volume was assessed in the patient’s space (i.e., prior to spatial normalization) by summing thresholded GM, WM and CSF probability maps (threshold = 0.2) followed by a count of non-zero voxels. Statistical values were corrected for multiple comparisons and thresholded at *p*_corrected_ < 0.05. Unthresholded corrected maps in nifti format are freely available for consultation and download at http://neurovault.org/collections/2466. Since we used whole-brain analysis, we verified that each significant cluster certainly arose from the tissue map of whom it depended so that white matter changes were not due to gray matter changes for instance. All clusters were anatomically defined according to the AAL atlas.

## Results

### Demographic data

Demographic data are presented in Table 1. According to results from ANOVA and post hoc analysis in the subset with prodromal patients (F[2,89] = 5.32; *p* < 0.01), the pro-AD group was significantly older than HC (*p* < 0.01) and we noted a trend compared to the pro-DLB group (*p* = 0.062), but the pro-DLB group did not differ from HC. No significant difference in gender was observed, but pro-AD patients were less likely to be female than pro-DLB patients (χ^2^, *p* = 0.066). The total intracranial volume did not differ between these three groups (ANOVA, F[2,89] = 0.08).

An age effect was observed in the subset with patients with dementia (ANOVA, F[2,62] = 6.26; *p* < 0.01): HC were younger than mild-DLB (*p* < 0.05) and mild-AD patients (*p* < 0.01) but mild-DLB patients did not differ from those with mild-AD. There was no gender effect. The total intracranial volume differed between groups (ANOVA, F[52,62] = 3.37; *p* < 0.05) since the mild-AD group had smaller values than the mild-DLB group (*p* < 0.05) but did not differ from HC. Total intracranial volume did not differ between mild-DLB patients and HC.

## VBM-DARTEL

### Prodromal patients

**Figure 1:**
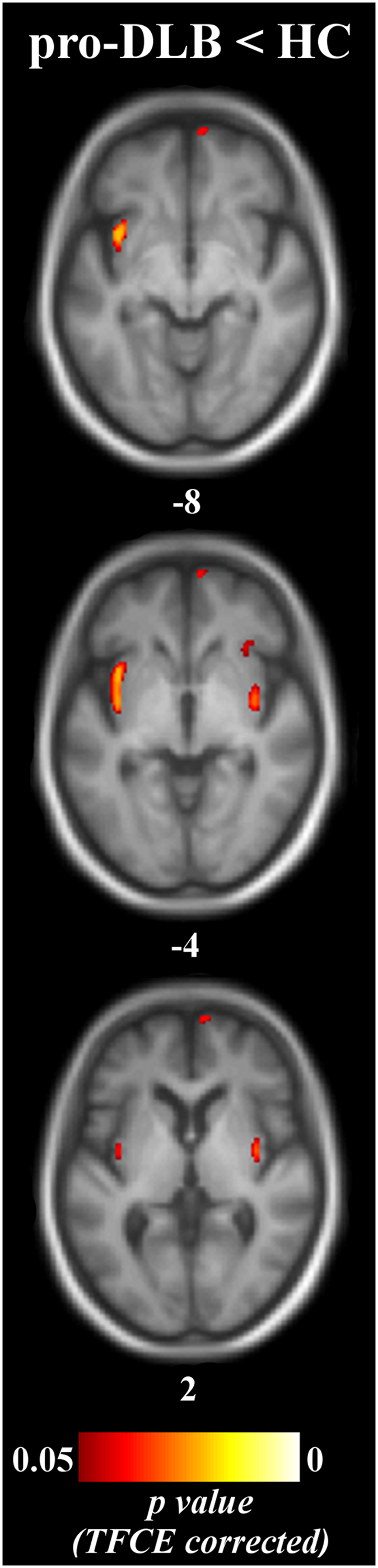
Patterns of significant gray matter loss across prodromal groups and healthy elderly controls pro-DLB, dementia with Lewy bodies at the prodromal stage; pro-AD, Alzheimer’s disease at the prodromal stage; HC, healthy controls. Results are expressed as *p*-values from permutation tests with threshold-free cluster enhancement corrected for multiple comparisons, and are superimposed on the mean MNI-standardized MRI T1-weighted image of the prodromal patients and healthy controls. Left is left side of the brain. Subscripts are the z-axis coordinates of the slice in the MNI space.

Unthresholded statistical images are freely available for consultation and download at http://neurovault.org/collections/2466 (the viewer has a threshold slider for which we recommend to set the left value to 0). Pro-DLB patients had a reduced GM volume in right medial frontal gyrus, and in bilateral insula and right claustrum relative to controls (Table 2, Fig. 1). Pro-AD patients did not differ from HC at *p*_corrected_ < 0.05, although a tendency towards a GM loss in right hippocampus was observed at a lower statistical threshold (*p*_corrected_ = 0.07 and *p*_uncorrected_ = 0.0002). Comparisons between prodromal groups did not reveal any decrease of GM volume. Controls did not show any atrophy that exceeded that in prodromal patients. The effect of age did occur either in the insula or in the medial temporal lobe.

**Table 2:** Anatomic location of the significant clusters revealing a loss of gray matter volume in the studied groups.

Contraste	Cluster	Laterality	Size in mm^3^	*p*-value	Coordinates (x, y, z)		
pro-DLB < HC	Medial frontal gyrus	R	1545	0.042	7	58	29
	(Superior and orbital parts)						
	Insula	L	1514	0.036	−38	10	−7
	Posterior insula – Claustrum	R	741	0.039	37	−4	−2
	Anterior insula	R	190	0.047	33	27	−2
mild-DLB < HC	Insula – Claustrum	L	2022	0.034	−35	−9	−5
mild-AD < HC	Parahippocampal gyrus	L	8613	0.023	−22	−5	−21
	Hippocampus – Amygdala						
	Putamen – Insula						
	Temporal pole						
	Parahippocampal gyrus	R	7306	0.020	24	−3	−21
	Hippocampus – Amygdala						
	Putamen – Insula						
	Temporal pole						
	Inferior temporal gyrus	L	3904	0.025	−56	−12	−30
	Inferior temporal gyrus	R	406	0.041	55	−29	27
	Fusiform gyrus	L	96	0.049	−29	−8	−36
	Superior temporal sulcus	L	76	0.049	−55	−12	−15
mild-AD < mild-DLB	Middle temporal gyrus	L	421	0.046	−56	−12	−30

pro-DLB, dementia with Lewy bodies at the prodromal stage; mild-DLB, dementia with Lewy bodies at the stage of dementia; pro-AD, Alzheimer’s disease at the prodromal stage; mild-AD, Alzheimer’s disease at the stage of dementia; HC, healthy controls. The clusters are named according to the Automated Anatomical Labeling template. Laterality: L, left hemisphere; R, right hemisphere. *P*-values are the mean corrected *p*-value in the cluster. Coordinates are in millimeters in the Montreal National Institute (MNI) space.

In terms of WM volume, no differences between groups were evidenced. The effect of age occurred in all lobes of the brain.

### Patients at the stage of dementia

**Figure 2:**
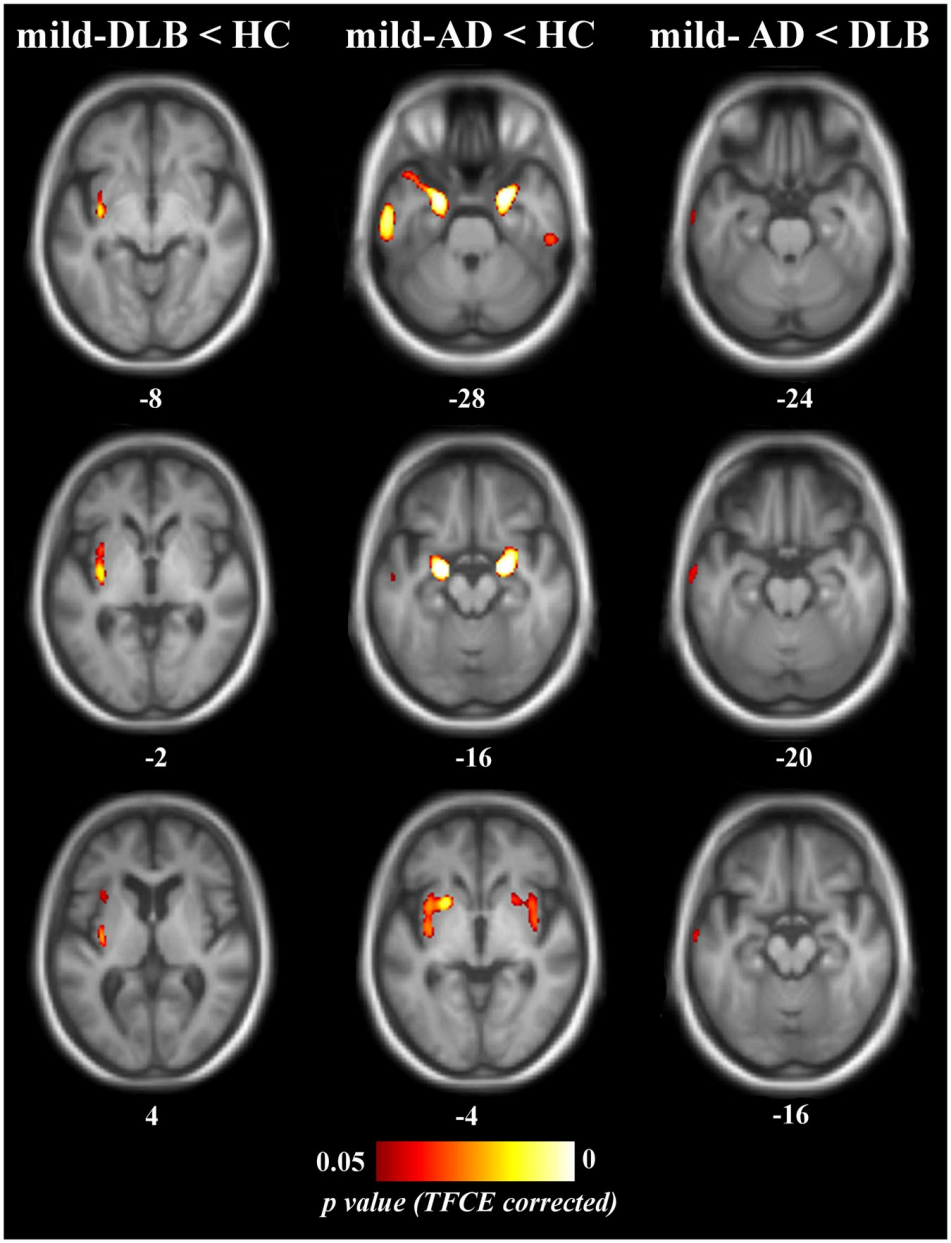
Patterns of significant gray matter loss across patient groups at the stage of dementia and healthy elderly controls mild-DLB, dementia with Lewy bodies at the stage of dementia; mild-AD, Alzheimer’s disease at the stage of dementia; HC, healthy controls. Results are expressed as *p*-values from permutation tests with threshold-free cluster enhancement corrected for multiple comparisons, and are superimposed on the mean MNI-standardized MRI T1-weighted image of the patients at the stage of dementia and healthy controls. Left is left side of the brain. Subscripts are the z-axis coordinates of the slice in the MNI space.

Unthresholded statistical images are freely available for consultation and download at http://neurovault.org/collections/2466 (the viewer has a threshold slider for which we recommend to set the left value to 0). Relative to controls, mild-DLB patients were atrophied in left insula and claustrum (Table 2, Fig. 2). In mild-AD patients compared to HC, the atrophy occurred extensively in parahippocampal gyrus, amygdala and hippocampal complex, temporal pole, but also insula and putamen, all bilaterally. In mild-AD patients relative to mild-DLB patients, left middle temporal gyrus was atrophied in mild-AD patients, whereas no significant differences were apparent in the mild-DLB group relative to the mild-AD group. In HC, no GM losses were apparent that exceeded that in patients at the stage of dementia. The effects of age did not occur either in the insula or in the medial temporal lobe.

**Figure 3:**
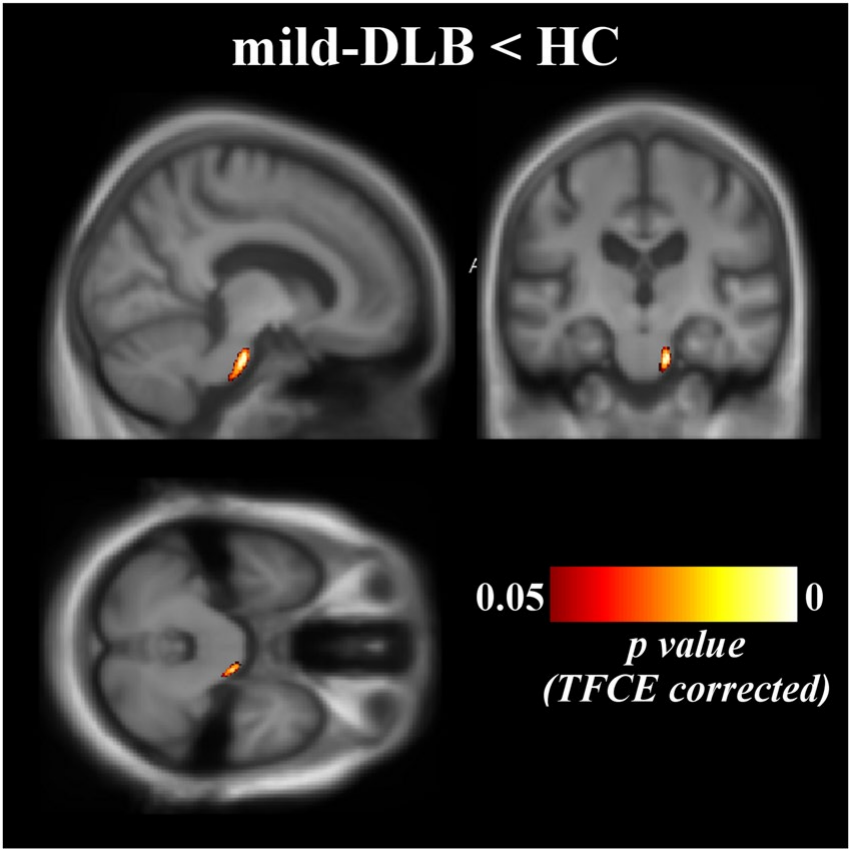
Patterns of significant white matter loss in mild-DLB patients mild-DLB, dementia with Lewy bodies at the stage of dementia; HC, healthy controls. Results are expressed as *p*-value from permutation tests with threshold-free cluster enhancement corrected for multiple comparisons, and are superimposed on the mean MNI-standardized MRI T1-weighted image of the patients at the stage of dementia and healthy controls. Left is left side of the brain. Coordinates of the multiplanar image in the MNI space: x = 11; y = −18; z = −30.

In terms of WM volume, mild-DLB patients had a loss of WM in the right part of the pons compared to HC (Fig. 3. Size in mm3: 556; mean *p*-value: 0.046; coordinates in the MNI space [x, y, z]: 8, −17, −29). No other differences reached statistical significance. The effect of age occurred in the frontal cortex.

## Discussion

Using VBM analysis, this study sought to assess patterns of GM atrophy in DLB at both prodromal and dementia stages compared to healthy elderly controls and patients with AD. Our assumptions were the followings: (1) pro-DLB patients would have GM atrophy predominantly in the anterior insula, but pro-AD patients would have GM atrophy in the entorhinal cortex and the hippocampus in such a way that patients group comparison would provide a similar pattern of atrophy; (2) at the stage of dementia, the GM atrophy of insula in DLB patients would enlarged to the entire insula, and expand to basal ganglia and frontal cortex, whereas the medial temporal lobe atrophy in AD patients would spread to insula, temporal, posterior cingulate and frontal cortices, and patients group comparison would provide a similar pattern of atrophy. If present, we also expected slight WM atrophy in the brainstem for prodromal DLB patients and in the medial temporal lobe for prodromal AD patients, which would enlarge at the stage of dementia. We report that early atrophy in DLB involved bilateral insula whereas it occurred rather in the medial temporal lobe in pro-AD (although at a lower statistical threshold). Comparisons of pro-DLB and pro-AD did not show differences in these structures. When the pathology worsened, the pattern of atrophy enlarged with deeper atrophy at the stage of mild dementia. Whereas in mild-DLB volume loss was observed in insula but not in hippocampus, mild-AD showed atrophy in both hippocampus and insula. Comparisons of patients with mild dementia revealed left middle temporal gyrus atrophy in AD relative to DLB.

The pattern of atrophy that we found in AD patients is consistent with previous studies reporting early atrophy of the medial temporal lobe, supporting as a consequence our hypotheses. The entorhinal cortex has been reported as reduced as early as the subjective cognitive impairment^13^, then the progression reached hippocampus in pro-AD^13–17^, and AD patients with dementia^14, 41^. Such losses of GM were shown to be a good predictor of cognitive decline and conversion to dementia^15, 18–21^. In addition to entorhinal cortex and hippocampus, we report a loss of gray matter in parahippocampal gyrus and amygdala in mild-AD, which has already been described in MCI^14, 21, 42^, although the atrophy occurred also in other brain structures known to be impaired in AD, such as the temporal pole, the inferior and superior temporal gyrus^23, 41, 43^, the fusiform gyrus^43^, but also the putamen and insula^43^.

Attesting to our hypothesis, atrophy in DLB mainly occurred in the bilateral insular cortex as early as the prodromal stage, and also included the claustrum. The deterioration of the insular cortices in DLB at the stage of dementia has been described in some previous studies^23, 25^. Moreover, a meta-analysis on GM atrophy in DLB patients at the stage of dementia found an atrophy of bilateral insula and basal ganglia^26^. However, we are the first to our knowledge to describe the occurrence of insular atrophy in DLB early in the development of the pathology. Moreover, the significant clusters were well localised and demonstrate that both the anterior and posterior parts of the insulae are impaired. This pattern of early atrophy does not support our assumption of an anteroposterior evolution of insular atrophy, although further longitudinal studies will provide new information about its. The topological distinction between anterior and posterior insula (sometimes subdivided into three: anterior, middle and posterior parts) was done according to cytoarchitectonic^44^ and histological^45^ analyses, task-based functional MRI^46^, functional connectivity MRI^47–49^, structural connectivity MRI^50–52^, or even multimodal imaging^53^. Briefly, these studies reported that anterior insula is connected to prefrontal and anterior cingulate cortices, whereas posterior insula is rather connected to motor cortex, superior temporal gyrus and the posterior parts of the brain. Accordingly, they also demonstrated that anterior insula is involved in emotional processes, saliency, attention and cognitive control, whereas posterior insula would be implicated in somatosensory and motor processing (including interoception and visceral motor, although other studies reported interoception involving the anterior insula^28, 54^). These data about insula are consistent with the known cognitive pattern of DLB at the early stage, including executive function impairment^55^ and attention deficit^8, 11, 56^. Moreover, stimulations of the anterior insula in epileptic patients were responsible for clinical responses including motor modifications such as tremor, hallucinations, and neurovegetative symptoms^28^. These exceptional clinical experiments are consistent with the known symptoms of DLB even at the prodromal stage^8^. In the same way, stimulation of a particular region between the anterior insula and the claustrum is responsible for impairment of consciousness, with staring, unresponsiveness, and slowing, and sometimes with confusion^57^, which are fully consistent with the known cognitive and alertness fluctuations in DLB. The claustrum, which is atrophied in the left hemisphere of mild-DLB patients together with the insula, is also involved in visual processing^58, 59^ and is connected to the occipital cortex^60, 61^, suggesting that the insula and the claustrum could be involved in the visual disorders in DLB^62^. According to our results and those of previous studies on DLB, and in view of the functions of the insula and its connections to other brain areas, the insula in DLB seems to be (1) affected at an early stage, (2) the main atrophy at the stage of dementia, and (3) related to the clinical profile of DLB, making the insula a key region for further investigations on DLB. Still, insula alone may not be all responsible for the disorders in DLB that we related to insula, but may play an essential role.

Compared to healthy controls, we found that atrophy in AD started with medial temporal lobe atrophy, then the pattern enlarged to other brain areas, including insula. In contrast, DLB started with insula atrophy and patients did not develop medial temporal atrophy, although we cannot rule out the possibility that more seriously affected DLB patients might have a decrease of GM volume in the medial temporal lobe^63^. In contrast to our assumptions, the comparison between DLB and AD did not revealed any loss of GM in AD in the medial temporal lobe but in the middle temporal gyrus, and no structure were more significantly atrophied in DLB compared to AD. Even though these results are in agreement with the clinical profile of patients, they do not support the hypothesis of a dissociation between an early atrophy of the medial temporal lobe in AD, which is followed by atrophy of the insula, and an early atrophy of the insula in DLB without loss of GM in the medial temporal lobe. While relevant, exploring this hypothesis is outside the scope of our study and will require longitudinal investigations. At the stage of dementia, both AD and DLB had atrophy of insula, and this might relates to the clinical symptoms that could be shared between DLB and AD.

Comparisons of WM volumes demonstrated only a reduction in the pons in mild-DLB compared to HC according to our hypothesis, which is in line with previous comparisons between DLB and AD^37^. Since this part of the brain stem is involved in arousal, autonomic functions, sleep, and acts as a relay between the cerebrum and the cerebellum, its atrophy would support some of the clinical signs of DLB, such as parkinsonism, Rapid Eye Movement sleep behavior disorder^11, 64^, dysautonomia and fluctuations^65^. Using fractional anisotropy or mean diffusion might provide additional information about WM changes in DLB, overcoming the restricted sensitivity of WM VBM analyses, and providing in addition a description of WM microstructural changes.

While the present results are consistent with the literature, there are still some limitations in our study. First, notwithstanding the statistical analyses accounting for the sample size, the groups are small, limiting the generalization of the results to a larger population. However, the cohort of pro-DLB is to our knowledge the largest to date in an MRI study. Second, age was not equally distributed across groups, even though we limited this effect by including it as a covariate in the statistical model. At the prodromal stage of DLB, patients did not differ in age from HC but were atrophied in insula. Moreover, no effect of age on GM and WM volumes were observed in insula and in the part of the pons which was atrophied in pro-DLB patients, respectively. Accordingly, the atrophies in DLB were not mistaken effects due to age but related to structural modifications due to DLB.

## Conclusion

This paper describes a loss of GM volume of bilateral insula in DLB, which started as early as the prodromal stage. According to the functions of the insula and its relationship with other brain areas, this atrophy is consistent with the clinical and cognitive profile of patients with DLB. In contrast, patients with AD were rather characterized by a decrease of GM in the medial temporal lobe, as described in several previous studies. Nevertheless, in contrast to our hypothesis, comparisons between DLB and AD revealed external temporal atrophy in AD. A potential dissociation between DLB and AD atrophy at the early stage, i.e. starting with either insula or medial temporal lobe, has still to be explored. Since the insula seems to play a key role in DLB, the repercussions of its impairment on the functioning of other brain areas, and on cognitive deficits as a consequence, should be further explored. Functional and structural connectivity, perfusion, and their correlations with cognitive performances could be such an approach.
